# Cone-beam computed tomography is not a mandatory procedure in adrenal venous sampling for primary hyperaldosteronism

**DOI:** 10.1186/s12880-022-00911-5

**Published:** 2022-11-03

**Authors:** Ran Cai, Chao Hu, Hai-Yang Li

**Affiliations:** Department of Radiology, The Second Xiangya Hospital of Central South University, No.139 Middle Renmin Road, Changsha, 410011 Hunan People’s Republic of China

**Keywords:** Adrenal glands, Cone-beam computed tomography, Adrenal venous sampling, Learning curve, Imaging pattern

## Abstract

**Objectives:**

To investigate the necessity of cone-beam computed tomography (CBCT) in adrenal venous sampling (AVS).

**Methods:**

This retrospective study included 120 consecutive patients with primary hyperaldosteronism who underwent AVS. Based on the learning curve of the interventional radiologists, the patients were divided into the learning (n = 36) and proficiency (n = 84) groups chronologically. Based on the imaging pattern of the right adrenal vein (RAV), the patients were divided into the typical (n = 36) and atypical (n = 84) groups. The success rate, radiation dose, and sampling time were compared among the entire study population and each subgroup.

**Results:**

A total of 69 patients underwent CBCT, whereas 51 patients did not. The overall success rate was 85.8%, and no difference was noted between patients with and without CBCT (*P* = 0.347). However, radiation dose (*P* = 0.018) and sampling time (*P* = 0.001) were significantly higher in patients who underwent CBCT than in patients who did not. In learning group, CBCT improved success rate from 62.5 to 96.4% (*P* = 0.028), whereas it was not found in the proficiency group (*P* = 0.693). Additionally, success rate in patients with an atypical RAV imaging pattern was significantly higher when CBCT was used than when it was not used (*P* = 0.041), whereas no difference was noted in patients with typical RAV imaging pattern (*P* = 0.511).

**Conclusion:**

For physicians not very experienced doing AVS, there is a clear significant improvement in success rate when CBCT is used. However, CBCT only has minimal benefit for experienced operators, meanwhile CBCT may take an extra time and increase the radiation dose during AVS.

## Introduction

Primary hyperaldosteronism (PA) accounts for secondary hypertension in more than 10% of patients, and the main causes of PA are aldosterone-producing adenoma (APA) and bilateral adrenal hyperplasia (BAH) [1–3]. Once the diagnosis of PA has been established, it is crucial to determine whether the cause of PA is APA or BAH because the management of the two is vastly different. As several guidelines recommend, adrenal venous sampling (AVS) is considered the gold standard for differentiating APA from BAH [4, 5]. However, the AVS technique is challenging, given a success rate of 60–90% [6, 7]. The main difficulty associated with AVS is the cannulation of the right adrenal vein (RAV), as it is often short and exhibits several anatomical variations [8–10]. In previous studies, cone-beam computed tomography (CBCT) was used, which may improve the overall success rate of AVS [11–17]. However, CBCT is a time-consuming procedure that requires additional radiation exposure. The following questions should be considered regarding the use of CBCT: In what situations should CBCT be performed? In what situations is CBCT not needed? Therefore, the present study aimed to investigate whether CBCT should be performed during AVS.

## Materials and methods

### Study population

The present study was retrospectively designed and performed at a single institution. From August 2015 to February 2021, 120 consecutive patients diagnosed with PA who underwent AVS were included. The study was approved by the institutional ethics committee in accordance with the Declaration of Helsinki. Due to the retrospective nature of the study, written informed consent was waived by the institutional ethics committee.

### Preprocedural CT imaging acquisition

All 120 patients underwent a preprocedural contrast-enhanced computed tomography scan. CT examination was performed using a single machine (SOMATOM Definition Flash, Siemens Healthineers). The CT acquisition protocols were as follows: spiral mode; supine position; inspiratory breath hold; rotation time, 0.5 s; and reconstructed slice thickness, 0.6–5 mm. An unenhanced CT scan was obtained through the adrenal glands prior to injection of the contrast agent. After the injection of contrast agent (Omnipaque, GE Healthcare), images of the arterial phase (25–35 s), portal venous phase (60–70 s), and delayed phase (15 min) were obtained. The position of the right adrenal gland was identified on axial and coronal multiplanar reformations.

### AVS procedure

The AVS procedure was initially performed in our institution in August 2015 by an AVS team, which was led by an interventional radiologist with 30 years of experience in vascular interventions using a digital subtraction angiography machine (IGS-530, GE Healthcare, USA). Antihypertensive medications that may interact with hormonal dosages were discontinued prior to the procedure. The bilateral common femoral veins were punctured under ultrasound guidance. A 6-F sheath and a 5-F sheath were then inserted into the bilateral common femoral veins. A 5-F Simmons II catheter was used to catheterize the left adrenal vein (LAV), and a 5-F MIK catheter or a 5-F Cobra catheter was used to catheterize the RAV. Contrast venography was also performed to delineate the bilateral adrenal veins. CBCT was performed with a manual injection or a power injector. The CBCT protocols were as follows: injection rate, 0.3–0.5 mL/s; delay exposure time, 6–8 s; total volume contrast agent injected, 2–4 mL; contrast agent to normal saline dilution ratio, 1:2; rotation time, 20°/s; and rotation degree, 200. Blood samples were simultaneously obtained from the RAV, LAV, and common femoral vein through a 6-F sheath 10 min after intravenous bolus injection of 250 µg of cosyntropin. Finally, 6 mL of blood sample for each vein was collected by aspiration with a syringe to measure the levels of cortisol and aldosterone.

### Data analysis

The cannulation success of AVS is defined as a bilateral selectivity index (SI) greater than 4 after adrenocorticotropic hormone stimulation, where SI is the ratio of adrenal vein cortisol to peripheral vein cortisol. After ensuring the success of sampling, the lateralization index (LI) was calculated. The LI is the ratio of the adrenal venous aldosterone/cortisol on the dominant side to the adrenal venous aldosterone/cortisol on the other side, and an LI greater than 4 indicates a lateralization of excess aldosterone production or unilateral disease. If unilateral lesions were visualized on CT and the dominant aldosterone secretion was lateralized on the same side, it was considered concordant with lateralization with preprocedural CT findings. In addition to the success rate, the radiation dose (assessed as the sum of the air kerma) and sampling time of each procedure were also documented. The sampling time was defined as the time interval between the beginning and the end of the procedure.

### Learning curved-based subgroups

AVS is a learning curve-based procedure that requires adequate experience to obtain a reliable success rate. The learning curve provided objective evidence on a case-by-case basis and showed changes in competence over time, aiming to objectively assess the number of cases in which surgeons acquire proficiency and trends in learning. A previous study demonstrated that 36 procedures served as a cutoff for AVS, and the success rate increased from 63 to 82% [18]. Therefore, the cutoff of 36 procedures was used to determine the learning and proficiency groups in a time order.

### Imaging pattern-based subgroups

**Fig. 1:**
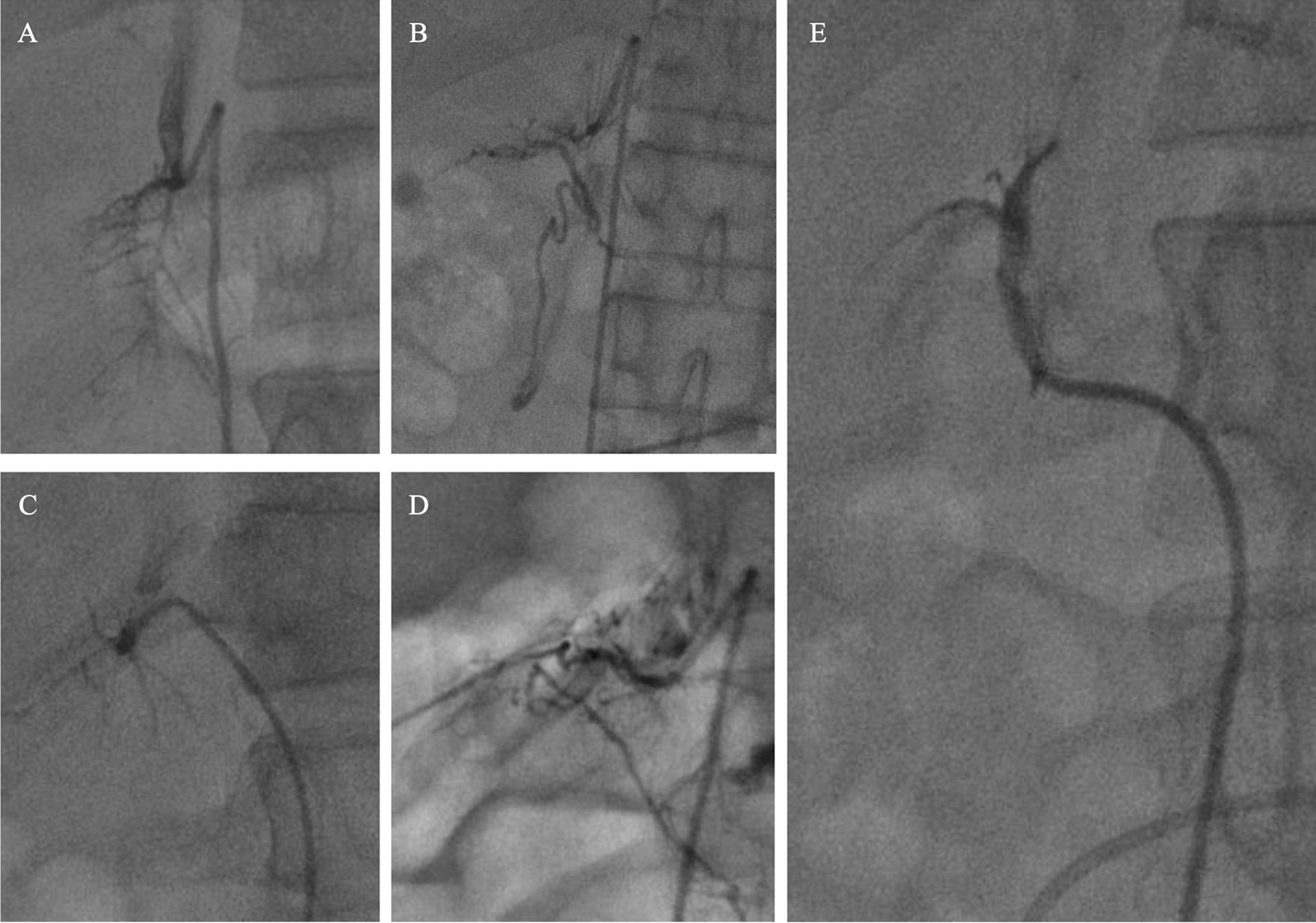
Imaging patterns of the right adrenal vein (RAV) on venography. A gland-like type of RAV with a main central stem and numerous branches (**A**); a triangular type of RAV with extensive communications (**B**); a spidery branch-like shape of RAV with communications (**C**); a delta type of RAV with fairly crowded vessels and extensive communications (**D**); an atypical type of RAV without an adrenal structure background (**E**)

As demonstrated in a previous study [19], typical RAV shapes include (1) a gland-like shape, (2) a delta shape with minimal filling of the internal structure, (3) a triangular shape with vessels fairly crowded together, and (4) a spidery branch-like shape. The atypical pattern of RAV includes (5) no discernible adrenal vessels, with only communicating veins present. In addition to these five shapes, RAV may communicate with the inferior vena cava (IVC), right renal vein, intercostal vein, and right phrenic vein through superficial or emissary veins. Figure 1 shows the imaging patterns of RAV.

In the present study, the RAV imaging pattern was divided into typical and atypical imaging patterns. The typical imaging pattern was a venography of RAV fulfilling both of the following: (1) a typical RAV shape and (2) the presence of communicating veins; otherwise, it was defined as an atypical imaging pattern.

### Statistical analysis

Continuous variables are presented as the mean and standard deviation, median with interquartile range (IQR), or frequency. Categorical variables were presented as numbers and percentages. Pearson’s chi-squared test or Fisher’s exact test was used to compare categorical variables, whereas the independent sample t-test or rank-sum (Mann–Whitney U) test was used to compare numerical variables. Commercially available software (SPSS version 20, International Business Machines Corporation) was used for all statistical analyses. Statistical significance was set at *P* < 0.05.

## Results

### Patients’ characteristics

**Fig. 2:**
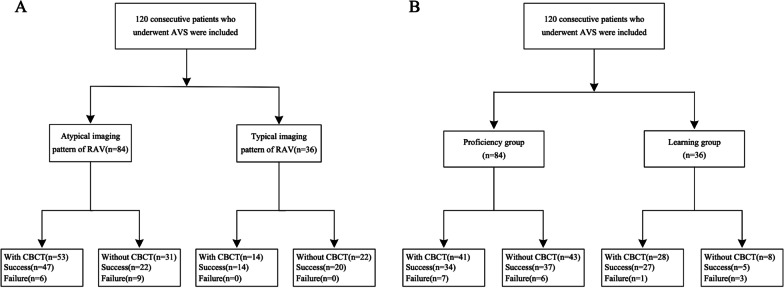
Flowchart of the study population for imaging pattern-based (**A**) and learning curve-based (**B**) classifications

A total of 120 patients (61 males and 59 females; mean age = 49.4 ± 10.3 years) were included in the study population. Among the participants, 69 underwent CBCT, whereas 51 did not undergo this procedure. There were 36 patients with typical RAV imaging patterns, whereas 84 patients had atypical RAV imaging patterns. According to the learning curve-based classification criterion, 36 patients from August 13, 2015 to June 26, 2018 were assigned to the learning group, and the remaining patients from July 5, 2018 to February 2, 2021 were assigned to the proficiency group. CBCT was more frequently performed in the learning group (28/36, 77.8%) than in the proficiency group (41/84, 48.8%) (*P* = 0.003). The baseline characteristics of patients in the entire study population are summarized in Table 1. A flowchart of the study population is presented in Fig. 2.

**Table 1:** The baseline characteristics of patients in the entire study population

Parameters	Overall (n = 120)	With CBCT (n = 69)	Without CBCT (n = 51)	*P*
Age (years)	49.4 ± 10.3	49.4 ± 11.2	49.9 ± 9.0	0.690^#^
Gender (%)				0.147*
Male	61 (50.8%)	39 (56.5%)	22 (43.1%)	
Female	59 (49.2%)	30 (43.5%)	29 (56.9%)	
Body mass index (kg/m^2^)	24.8 (IQR: 4.1)	25.8 ± 4.2	24.5 ± 2.7	0.062^#^
Lowest kalemia (mmol/l)	3.3 (IQR: 0.8)	3.4 (IQR: 0.8)	3.3 ± 0.5	0.273^^^
Systolic blood pressure (mm Hg)	157.1 ± 21.6	158.8 ± 21.2	154.8 ± 22.1	0.316^#^
Diastolic blood pressure (mm Hg)	96.1 ± 14.8	95.7 ± 14.9	96.6 ± 14.8	0.749^#^
Plasma supine Aldosterone (ng/dl)	28.8 (IQR: 36.9)	29.7 (IQR: 23.2)	24.6 (IQR: 44.8)	0.574^^^
Plasma upright Aldosterone (ng/dl)	31.9 (IQR: 31.6)	32.3 (IQR: 29.1)	28.8 (IQR: 37.9)	0.804^^^
Plasma supine active renin (ng/ml.h)	0.5 (IQR: 1.1)	0.6 (IQR: 1.0)	0.5 (IQR: 1.3)	0.902^^^
Plasma upright active renin (ng/ml.h)	0.8 (IQR: 1.8)	0.8 (IQR: 1.7)	0.9 (IQR: 2.0)	0.706^^^
Aldosterone-to-renin ratio	44.7 (IQR: 97.6)	44.7 (IQR: 72.2)	52.2 (IQR: 125.1)	0.512^^^
Success rate (%)	85.8%	88.4%	82.3%	0.347*
Radiation dose (mGy)	617.0 (IQR: 784.2)	694.0 (IQR: 940.5)	490.0 (IQR: 718.0)	0.018^^^
Sampling time (min)	93 (IQR: 40.0)	101 (IQR: 44)	87.3 ± 32.0	0.001^^^

*CBCT* Cone-beam computed tomography;*IQR* Interquartile range

*Pearson’s chi-square test; ^#^One-Way ANOVA test; ^^^ Mann–Whitney U test

### Success rate

The overall success rate of AVS was 85.8% (103/120), with an individual success rate of 85.8% (103/120) for the right side and 97.5% (117/120) for the left side. In addition, 17 patients failed AVS, including one patient with ruptured RAV during CBCT and 16 patients with incorrect identification of the adrenal vein or who dropped off the catheter during AVS. The concordance rate of preprocedural CT imaging and the AVS procedure was 41.7% (50/120), while the discordance rate was 44.2% (53/120). The outcomes of AVS in the present study population are summarized in Table 2. No difference was noted in the success rates between patients with and without CBCT (88.4% vs. 82.3%, *P* = 0.347).

**Table 2:** The outcomes of AVS in the entire study population

	Overall (n = 120) (%)	With CBCT (n = 69) (%)	Without CBCT (n = 51) (%)
*SI*			
Right (≥4/%)	103/85.8	61/88.4	42/82.3
Left (≥4/%)	117/97.5	68/98.5	49/96.1
Bilateral (≥4/%)	103/85.8	61/88.4	42/82.3
*LI*			
Right (≥4/%)	31/25.8	24/34.8	7/13.7
Left (≥4/%)	31/25.8	19/27.5	12/23.5
Bilateral (< 4/%)	41/34.2	18/26.1	23/45.1
AVS-CT concordance (%) *l			
Concordant	50/41.7	32/46.4	18/35.3
Discordant	53/44.2	29/42.0	24/47.1

*AVS* Adrenal venous sampling;* CBCT* Cone-beam computed tomography;* SI* Selectivity index;* LI* Lateralization index;* CT* Computed tomography

*AVS-CT concordance: unilateral lesions on CT and ipsilateral lateralization on AVS or bilateral lesions on CT and no lateralization on AVS; AVS-CT discordance: unilateral lesions on CT and contralateral or no lateralization on AVS

**Fig. 3:**
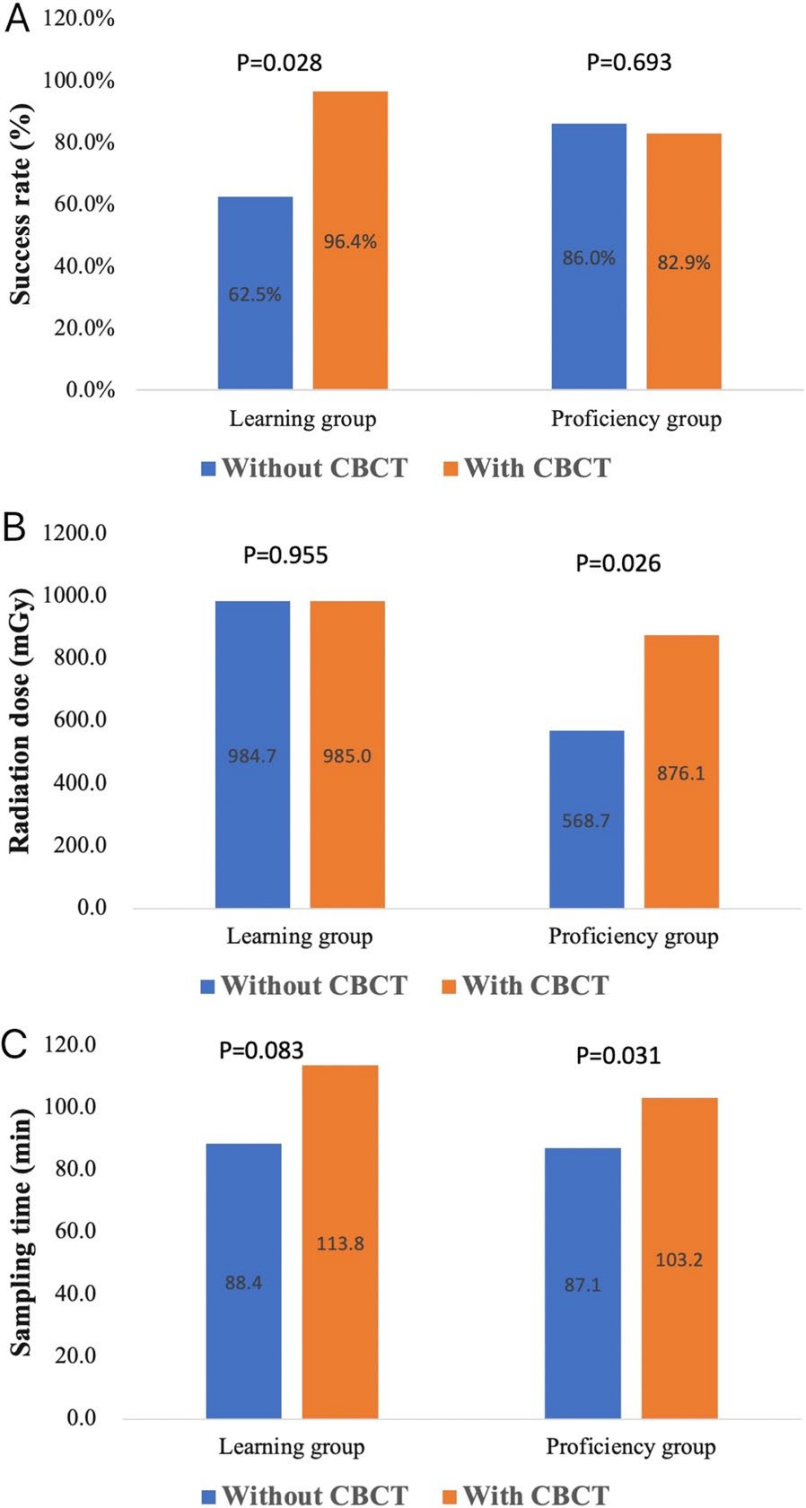
Histograms of the success rate (**A**), radiation dose (**B**), and sampling time (**C**) in the learning and proficiency groups

**Fig. 4:**
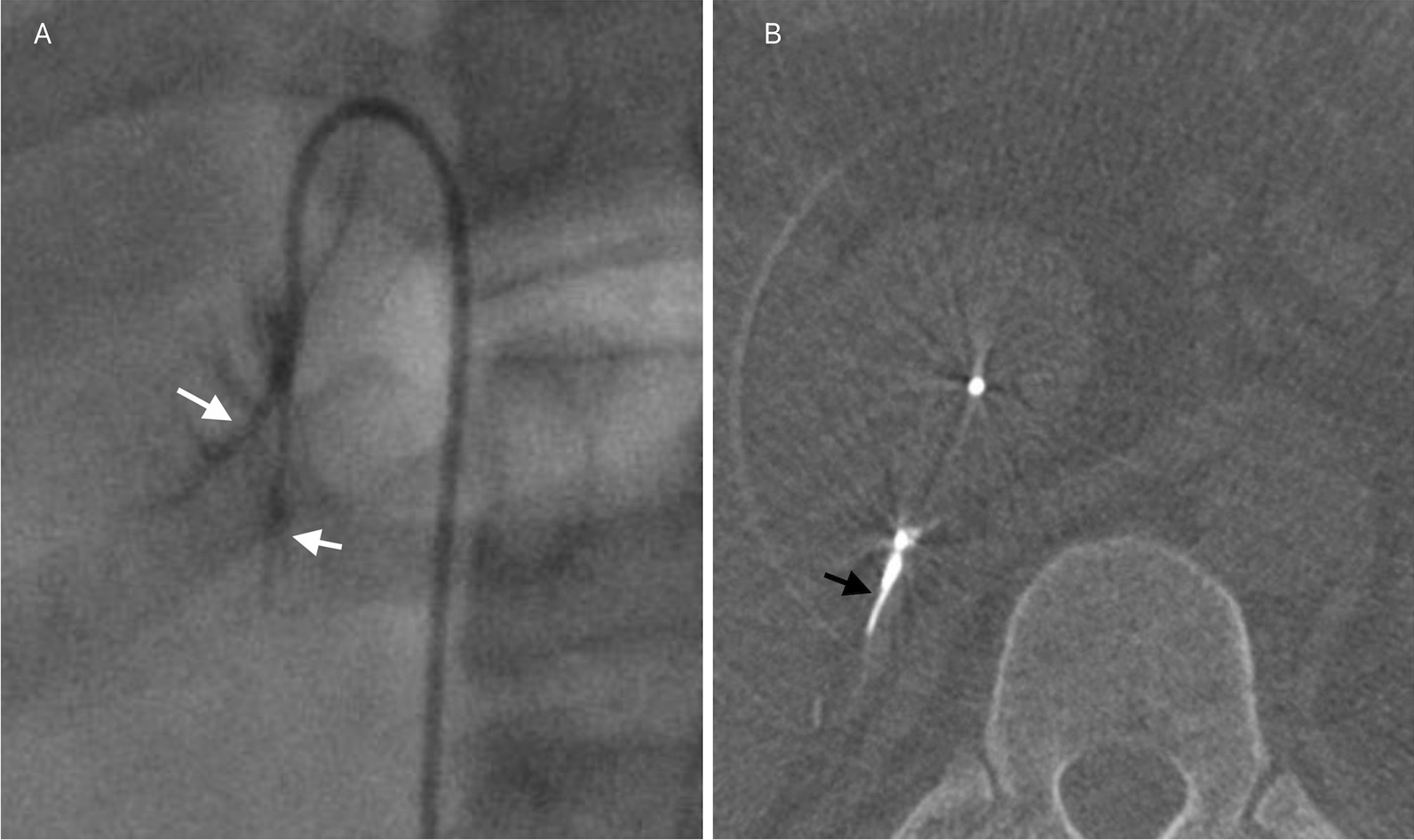
A 30-year-old male with primary hyperaldosteronism who underwent adrenal venous sampling (AVS). The right-side venogram showed a delta type (white arrow) of RAV without communications that was classified into the atypical imaging pattern group (**A**). CBCT was performed, and RAV was confirmed (black arrow) (**B**)

In the learning group, there was a significant improvement in the success rate of AVS when performed with CBCT, increasing from 62.5% (5/8) to 96.4% (27/28) (*P* = 0.028). However, this improvement was not observed in the proficiency group (*P* = 0.693), with a success rate of 82.9% (34/41) in patients who underwent CBCT and 86.0% (37/43) in patients who did not (Fig. 3A). In patients with a typical RAV imaging pattern, CBCT might not improve the success rate (*P* = 0.511) of AVS. However, in patients with atypical RAV imaging patterns, a significant success rate was noted in patients with CBCT than in those without CBCT (*P* = 0.041). The detailed information of the success rate in patients with typical and atypical imaging pattern of RAV was illustrated in Table 3. Moreover, CBCT may improve the success rate of AVS from 50 to 95.2% in patients with atypical RAV imaging patterns (*P* = 0.006) in the learning group, while this improvement was not observed in patients with typical RAV imaging patterns. In the proficiency group, CBCT might not improve the success rate in patients with typical (*P* = 0.385) and atypical (*P* = 0.427) RAV imaging patterns. Figure 4 shows a patient in the learning group with an atypical RAV imaging pattern, which the RAV is confirmed by CBCT.

**Table 3:** Characteristics of patients with typical or atypical imaging patterns of RAV

Parameters	Typical imaging pattern (n = 36)			Atypical imaging pattern (n = 84)		
	With CBCT (n = 14)	Without CBCT (n = 22)	*P*	With CBCT (n = 53)	Without CBCT (n = 31)	*P*
Success rate (%)	100.0	90.9	0.511`	88.7	71.0	0.041*
Radiation dose (mGy)	801.7 ±663.4	604.9 ± 465.8	0.302^#^	694.0 (IQR: 805.5)	490.0 (IQR: 819.0)	0.050^^^
Sampling time (min)	117.0 ± 27.1	93.4 ± 28.6	0.019^#^	94.0 (IQR: 31.5)	87.1 ± 37.3	0.049^^^

*RAV* Right adrenal vein;* CBCT* Cone-beam computed tomography;* IQR* Interquartile range

`Fisher’s exact test; *Pearson’s chi-square test; ^#^One-Way ANOVA test; ^^^Mann–Whitney U test

### Radiation dose

The median radiation dose for the entire study population was 617.0 mGy (IQR: 784.2 mGy), which was significantly higher in patients with CBCT than in patients without CBCT (*P* = 0.018). In the learning group, there was no difference in the radiation dose between patients with and without CBCT (*P* = 0.955), while this difference was noted in the proficiency group, in which the radiation dose was significantly higher in patients with CBCT than in patients without CBCT (*P* = 0.026) (Fig. 3B). Regarding the imaging pattern of RAV, both patients with typical (*P* = 0.302) and atypical (*P* = 0.050) RAV imaging patterns showed no differences in radiation dose between patients with and without CBCT. The detailed information of the radiation dose in patients with typical and atypical imaging pattern of RAV was illustrated in Table 3.

### Sampling time

The median sampling time was 93 min (IQR: 40.0 min) in the entire study population, and it was significantly higher in patients with CBCT than in patients without CBCT (*P* = 0.001). In the learning group, there was no difference in sampling time between patients with and without CBCT (*P* = 0.083), whereas it was significantly higher in patients with CBCT than in patients without CBCT (*P* = 0.031) (Fig. 3C). Regarding the imaging pattern of RAV, both patients with typical (*P* = 0.019) and atypical (*P* = 0.049) RAV imaging patterns showed a higher sampling time in patients with CBCT than in patients without CBCT. The detailed information of the sampling time in patients with typical and atypical imaging pattern of RAV was illustrated in Table 3.

## Discussion

The value of CBCT during AVS has long been investigated, and most studies have suggested that CBCT is a useful tool for identifying RAV, thereby improving the success rate of AVS [11–17]. However, the present study showed contrasting results, in which a similar success rate was noted in patients with or without the assistance of CBCT (88.4% vs. 82.3%, *P* = 0.347).

Based on this contrary finding, the following questions were raised regarding the AVS procedure: In what situations should CBCT be performed? In what situations is CBCT not needed? In the present study, CBCT was mostly performed in the learning stage to confirm the catheterization of the RAV, and the results showed that CBCT could improve the success rate of AVS from 62.5 to 96.4% in this stage (*P* = 0.028). Therefore, it is hypothesized that CBCT may be helpful for interventional radiologists who are in the initial stages of learning to perform AVS. In addition, the present study also showed that CBCT could not improve the success rate of AVS when performed by an experienced interventional radiologist, suggesting that CBCT may not be needed in this situation.

However, the learning curve for AVS was based on personality traits. Some individuals may learn faster, whereas others may require more practice. In addition to the learning curve, the present study showed that the success rate could be improved from 71.0 to 88.7% with the assistance of CBCT in patients with atypical RAV imaging patterns. However, in patients with typical RAV imaging patterns, the success rate did not increase despite the assistance of CBCT. Thus, it is hypothesized that CBCT may be helpful in patients with atypical RAV imaging patterns and may not be needed in patients with typical RAV imaging patterns.

A previous study described various imaging appearances of RAV on venography [19]. According to the imaging appearance described, a classification criterion was established in the present study to divide the RAV imaging pattern into typical or atypical categories [19]. With this classification, RAV on venography can be accurately identified even without the help of CBCT. To the best of our knowledge, this is the first study to clarify that patients with atypical RAV imaging patterns will benefit from CBCT when performing AVS. RAV has various shapes and variations in venography, which is the main difficulty in performing AVS. As it is occasionally improperly identified, the shape of right-side venous injection may not be a reliable feature for recognizing the RAV [8, 9, 17]. In addition to the venous shape, the presence of communications with the IVC, right renal vein, intercostal vein, and right phrenic vein through superficial or emissary veins is one of the most important imaging features for the identification of RAV [19]. In the present study, there was a high confidence level for the identification of RAV when venous injection showed the typical type of RAV accompanied by communication. In addition, the results showed that the radiation dose (*P* = 0.018) and sampling time (*P* = 0.001) were significantly higher in patients with CBCT than in patients without CBCT in the overall study population. Radiation dose is also a significant concern when performing AVS. Due to the additional exposure, the radiation dose is increased in a single patient, suggesting that CBCT should be performed cautiously [20].

Furthermore, one patient experienced RAV rupture during CBCT in the present study, which led to the failure of AVS. Because the RAV is usually small, the injection pressure may be too high when using a power injector, which may burst the RAV. Therefore, when performing CBCT, a manual injection technique is preferred over a power injector.

There are several limitations to the present study. First, this was a retrospective study with a relatively small number of patients included, and thus may be subject to selection and statistical bias. Second, the on-site quick cortisol assay (QCA) was not used in the present study, despite its use in improving the success rate, as reported in a previous study [21]. However, QCA is not available in many centers; therefore, it has limited use in clinical practice. Third, CBCT was performed only in the determination of RAV, while several other causes may influence the success rate of AVS, such as cannulation of the LAV since LAV also has many variations [2]. Finally, this study was performed in a single center, and the imaging classification criteria for identifying RAV should be validated in other centers.

In conclusion, CBCT is not a mandatory and should be cautiously used procedure for experienced operators during AVS, unless there are uncertainties about definite catheterization of the RAV or the venous anatomy is very atypical. However, less experienced operators may benefit from CBCT, since they are naturally less confident and need confirmation by CBCT before proceeding to sampling.

## **Main points**

1. Cone-beam computed tomography is helpful for an interventional radiologist in the beginning stages of learning how to perform adrenal venous sampling.
2. Cone-beam computed tomography is helpful in patients with atypical imaging pattern of right adrenal vein.
3. Cone-beam computed tomography may increase the additional radiation dose and sampling time when performing adrenal venous sampling.

## Data Availability

The datasets generated and/or analysed during the current study are not publicly available due data protection but are available from the corresponding author on reasonable request.
